# The Air-Breathing Paradise Fish (*Macropodus opercularis*) Differs From Aquatic Breathers in Strategies to Maintain Energy Homeostasis Under Hypoxic and Thermal Stresses

**DOI:** 10.3389/fphys.2018.01645

**Published:** 2018-11-21

**Authors:** Min-Chen Wang, Hui-Chen Lin

**Affiliations:** Department of Life Science, Tunghai University, Taichung, Taiwan

**Keywords:** energy allocation, metabolism, ionoregulation, Na^+^/K^+^-ATPase, air-breathing respiration

## Abstract

Two major strategies are used by most fish to maintain energy homeostasis under hypoxia. One is to utilize alternative metabolic pathways to increase energy production, and the other is to limit energy expenditure by suppressing energy-consuming processes, especially ionoregulation. Some anabantoid fishes live in tropical rivers, where hypoxic environments occur frequently. We previously found that under ambient hypoxia, anabantoid fishes do not downregulate Na^+^/K^+^-ATPase (NKA) activity to conserve energy in gills but instead increase the frequency of air-breathing respiration (ABR). In addition to the hypoxic condition, another factor that may cause cellular hypoxia in fish is abnormally high environmental temperatures. The frequency of such extreme thermal events has increased due to global climate change. In the present study, we examined whether the anabantoid fish, *Macropodus opercularis* employs the two strategies mentioned above to resist both ambient hypoxic and elevated thermal (cellular hypoxic) conditions. Results indicate that neither glucose metabolism nor gill NKA activity were altered by hypoxia (DO = 1.5 ± 1 mg/L), but glucose metabolism was increased by thermal stress (34 ± 1°C). NH_4_^+^ excretion and ABR frequency were both increased under hypoxia, thermal or hypoxic-and-thermal treatments. In fish that were restricted from breathing air, increased mortality and glucose metabolism were observed under hypoxic or thermal treatments. These results suggest that for *M. opercularis*, increasing ABR is an important strategy for coping with unmet oxygen demand under hypoxic or thermal stress. This behavioral compensation allows anabantoid fish to physiologically withstand hypoxic and thermal stresses, and constitutes a mechanism of stress resistance that is unavailable to water-breathing fishes.

## Introduction

The availability of oxygen is directly related to the capacity for energy production that drives metabolic processes. However, in fish, abnormally high temperatures cause increased metabolic energy expenditure (Richards et al., 2009), leading to an increased oxygen demand. As aerobic ectotherms, fish evolves strategies to overcome low levels of dissolved oxygen (DO) in the aquatic environment or temperature-induced elevations of oxygen demand. In several previous studies, different coping strategies were reported when fish were separately exposed to these two stresses (Zhou et al., 2000; Cooper et al., 2002; Costas et al., 2011), but few studies have examined the effects of thermally induced cell hypoxia or simultaneous hypoxic and thermal stress on fish.

During hypoxia, fish frequently use two physiological strategies to minimize energy demand and maintain energy homeostasis. The first strategy is to meet energy production requirements by adjusting flux through metabolic pathways, while the other is to reduce energy demand (Bickler and Buck, 2007; Richards et al., 2009). Glucose, the fuel of carbohydrate metabolism, is produced by glycogenolysis from glycogen store (Moyes and Schulte, 2008). Glucose participates into circulation for energy fuel (ATP) production by aerobic or anaerobic metabolic pathways (Moyes and Schulte, 2008). The rate-limiting first step of glycogenolysis is performed by glycogen phosphorylase (GP), which releases glucose-1-phosphate from the glycogen molecule (Moyes and Schulte, 2008). According to previous studies, glucose used in energy metabolism related to fish hypoxic-resistance (Chippari-Gomes et al., 2005; Richards et al., 2009). High mobilization of glucose from liver or muscle enhanced use of exogenous glucose in high energy-consumption organ (Chippari-Gomes et al., 2005; Tseng et al., 2007). Gill has glycogen-rich (GR) cells, which are adjacent to mitochondrion-rich (MR) cells (high energy-consuming ionocytes) in the gills (Evans et al., 2005; Tseng et al., 2008; Hwang et al., 2011). During times of acute metabolic stress, lactate is formed when glucose served as an emergency energy fuel and is transported form GR cells to the adjacent MR cells via monocarboxylate transporter before the liver degrades its reserve glycogen (Chang et al., 2007; Tseng et al., 2007; Tseng and Hwang, 2008; Hwang et al., 2011). In addition to carbohydrate metabolism, fish metabolize ingested proteins into amino acids, which are utilized as an energy source for producing ATP and NH_4_^+^ (Nelson and Cox, 2007; Barnes et al., 2011). On the other hand, NH_4_^+^ is a toxic substrate and cannot accumulate in the body. Thus, it is excreted in the form of NH_3_ and H^+^ and may again become NH_4_^+^ outside the body. However, it has been shown that fish slow down amino acid metabolism to reduce metabolic costs of ammonia excretion under hypoxic stress (Wood et al., 2007).

In order to limit energy expenditure during hypoxic stress, fish may decrease energy demand by reducing both physical movements and ion transport. The Na^+^/K^+^-ATPase (NKA) is a universal membrane-bound enzyme that provides the driving force for ion transport and intracellular homeostasis (Evans et al., 2005; Hwang et al., 2011). Thus, NKA activity is often used as an indicator for ion channel arrest (Huang et al., 2015a). In water-breathing fishes, hypoxia-tolerant species often decrease branchial NKA activity under hypoxic stress (Wood et al., 2007; Lundgreen et al., 2008; Richards et al., 2009). In contrast, hypoxia-intolerant fish, such as *Oncorhynchus mykiss*, do not reduce energy demand by suppressing NKA activity under hypoxic stress (Iftikar et al., 2010), but, instead, it enhanced glycogen degradation. The ability to modulate NKA activity can be a key difference between hypoxia-tolerant and intolerant fishes.

In the hypoxia-tolerant air-breathing anabantoid fish, branchial NKA expression was found to be unaltered by hypoxic stress (Huang et al., 2015b,c). Instead, the air-breathing respiration (ABR) frequency of anabantoid fish was increased to satisfy oxygen demand during hypoxia. The anabantoid fish, *Macropodus opercularis* of South and Southeast Asia, is not only hypoxia tolerant, but it is also a thermal-tolerant species that occupies natural ponds, minor creeks and farm waters, where DO levels and water temperatures may change rapidly (Graham, 1997; Wang et al., 1999). In this study, we investigated the strategies utilized by *M. opercularis* to maintain energy homeostasis during hypoxic, thermal or hypoxic-and-thermal stresses from gill to whole body physiological mechanism. We examined branchial lactate concentration, GP protein expression and NKA activity to understand the energy homeostasis mechanisms in gill. Second, ABR frequency and glucose and NH_4_^+^ concentrations were measured for increasing our knowledge of energy source usage of *M. opercularis* in whole body scale. Otherwise, we also evaluated whether air breathing behavior is critical for hypoxic and thermal tolerance in *M. opercularis*, and whether the physiological responses of this air-breathing species are different from those reported for most water-breathing fish.

## Materials and Methods

### Experimental Design

*Macropodus opercularis* were obtained from a local commercial source. Fish were fed daily with commercial fish food (NOVO Bits, JBL, Germany) and acclimated to aerated local tap water and a 12L:12D photoperiod in a 140-l plastic tank for more than 1 week prior to experimentation. The temperature and dissolved oxygen (DO, measured by a Horiba, DO sensor 5401, Japan) were controlled at 27.6 ± 0.4°C and 5.20 ± 0.52 mg/l, respectively. The average standard body length and weight of experimental fish are showed in Tables 1, 2. One day before the experiment, 24 acclimated fish were randomly assigned to four tanks with 2 L of aerated local tap water as experimental tank. Before the experiment, fish were fasted for 24 h. At every sampling time, two fish were anesthetized with MS-222 (0.4 mg/ml, 3-aminobenzoic acid ethyl ester, Sigma, St. Louis, MO, United States), respectively. Gills were sampled at 4°C and stored at −80°C. For independent sampling, each individual was collected for no more than two physiological parameters. For example, when we collected blood glucose samples from 10 individuals, we collected the gills of the first five fish for NKA activity and the last five ones for detecting GP.

**Table 1:** Water parameters, fish length and weight, and mortality in control (C), hypoxic (H), thermal (T), and hypoxic-and-thermal (HT) treatment ABR groups.

Treatment	Dissolved oxygen of water (mg/L)	Temperature of water (°C)	Body length (cm)	Body weight (g)	Mortality (%)
C	5.44 ± 0.47	27.37 ± 0.45	4.62 ± 0.91	3.69 ± 0.31	0 (0/145)
H	1.42 ± 0.31	26.32 ± 0.34	4.61 ± 0.83	3.66 ± 0.25	0 (1/143)
T	5.02 ± 0.42	34.47 ± 0.24	4.53 ± 0.85	3.60 ± 0.22	2.24 (3/133)
HT	1.2 ± 0.55	33.94 ± 0.55	4.48 ± 0.89	3.51 ± 0.10	4.88 (6/123)

**Table 2:** Water parameters, fish length and weight, and mortality in control (C), restricted (R), hypoxic (HR), and thermal (TR) treatment non-ABR groups.

Treatment	Dissolved oxygen of water (mg/L)	Temperature of water (°C)	Body length (cm)	Body weight (g)	Mortality in 10 min (%)	Mortality in 20 min (%)
C	4.94 ± 0.35 ^∗1^	27.17 ± 0.45	4.63 ± 0.35	3.77 ± 1.28	0 (0/80) ^∗2^	0 (0/18)
R	5.37 ± 0.23	27.25 ± 0.21	4.47 ± 0.94	3.59 ± 1.12	0 (0/76)	0 (0/8)
HR	1.29 ± 0.35	27.12 ± 0.35	4.31 ± 0.43	3.57 ± 1.10	0 (0/41)	100 (8/8)
TR	5.14 ± 0.47	34.31 ± 0.43	4.24 ± 0.12	3.67 ± 1.28	0 (0/48)	100 (8/8)

*^∗1^ All values are presented as average ± SEM; ^∗2^ the numbers inside the parenthesis shows number of dead fish/total fish the study used in each treatment.*

In the air-breathing group, fish were subjected to the control (C), hypoxic (H), thermal (T), and hypoxic-and-thermal (HT) treatments. Air-breathing fish were sampled at 2 h and 6 h after exposure to each treatment. In the ABR-restricted group, the fish were subjected to the control (C), restricted (R), hypoxic-and-restricted (HR), and thermal-restricted (TR) treatments. Since we had a 100% mortality (Table 2) at 20 min after fish were transfer to HR and TR treatments, *M. opercularis* that was exposed to HR and TR treatments were sampled 10 min after exposure. Fish exposed to C and R treatments were sampled at 10 min, 2 h and 6 h after exposure.

Each experimental tank had air stone for air input to maintain DO level and electrical heater (Tzong Yang Aquarium Company, Tainan, Taiwan) for temperature control. For hypoxic treatment, the DO in the water was controlled by gently sparging N_2_. For treatment groups where air breathing was restricted, a submerged plastic board was used to cover the tanks and prevent fish from reaching the water surface. The water parameter and fish mortality are summarized in Tables 1, 2. Experiments commenced at 9:30 a.m. Control fish were maintained in the same tank as they were fasted in and were harvested at the same times as treated fish.

### Air-Breathing Frequency

The air-breathing frequency was recorded by video cameras (DCR-HC 46, Sony, Japan) for 1 h in the control group (0 h). After the fish were transferred to their respective treatment tanks, air-breathing frequencies were recorded 0.5 h before the sampling time and lasted for another 0.5 h. The air-breathing frequency was defined as the number of times per min that the fish swam to the water surface and gulped air within the 60 min recording period.

### Blood Glucose Concentration

Fish was sacrificed by spinal transaction and 1.5 μl of blood collected for measured blood glucose concentration by the method developed by Tang et al. (2014) using a blood glucose monitor (ACCU-CHEK GO, Roche, Mannheim, Germany).

### Gill Lactate Concentration

Gills were homogenized (TissueLyser, Qiagen, Germany) in lactate assay buffer for 1 min and centrifuged at 10,000 × *g* for 10 min at 4°C. The supernatant was collected for testing. The gill lactate concentration was determined with a BioVision kit (Lactate colorimetric assay kit II, BioVision, Milpitas, CA, United States).

### Total Protein Extraction and Quantification

Gills were homogenized (TissueLyser, Qiagen, Germany) for 1 min with homogenizing medium (100 mM imidazole, 5 mM Na_2_EDTA, 100 mM sucrose, 0.1% sodium deoxycholate) and protease inhibitors (3.31 mM antipain, 2.16 mM leupeptin and 1.92 M benzamidine) in an aprotinin saline solution (5–10 trypsin inhibitor units/ml, Sigma, St. Louis, MO, United States). Homogenates were centrifuged at 4°C (EBR12R, Hettich, Germany) to obtain supernatants (total protein). Total protein concentration was measured by a spectrophotometer (U-2001, Hitachi, Japan) at a wavelength of 595 nm with bovine serum albumin (BSA) as the standard. The standard concentrations were 0, 2, 4, and 8 mM.

### Western Blot

Each protein sample was mixed with 4× sample-loading buffer [20 mM Tris-HCl, pH 6.8, 8% sodium dodecyl sulfate (SDS), 1 mM dithiothreitol, 40% glycerol, and 0.4% bromophenol blue]. The denatured protein samples were separated on SDS-polyacrylamide gels. After electrophoresis, protein samples were transferred to polyvinylidene difluoride membranes (PVDF membrane, NEN LIFE Science, Boston, MA, United States). The blotted membranes were blocked with BSA in PBST buffer (136.9 mM NaCl, 2.68 mM KCl, 6.39 mM Na_2_HPO_4_⋅2H_2_O, 1.76 mM KH_2_PO_4_ and 0.5% Tween 20, pH 7.4) at room temperature. The blotted membranes were incubated with primary antibody for 2 h at room temperature and then incubated with secondary antibodies. The proteins of interest were detected by the Chemiluminescence Reagent Plus system (NEN Life Science). The relative protein abundance was detected and photographed with an Intelligent Dark Box II with a Fujifilm LAS-1000 camera and images were analyzed by Image Gauge 4.0 (Fujifilm).

Mouse anti-human GPBB monoclonal antibody (mAb) (Biotrend Chemilalien GmbH, Cologen, Germany), and mouse anti-chicken β-actin mAb (Millipore, Darmstadt, Germany) were used as primary antibodies. Secondary antibodies included alkaline phosphate (AP) conjugated goat anti-mouse immunoglobulin G (IgG) (Jackson ImmunoResearch, West Grove, PA, United States). The relative protein abundance of GPBB was normalized to β-actin.

### Concentration of Excreted NH_4_^+^

The NH_4_^+^ concentration was determined in a microplate according to the method of Holmes et al. (1999). Water samples were collected directly from the experimental tanks and stored at 4°C. Each sample was mixed with working reagent (21 mM sodium tetraborate, 0.063 mM sodium sulfite, and 50 ml/l OPA in ethanol: 4 g of OPA in 100% ethanol). The solution was mixed by shaking and allowed to stand for 2.5 h in the dark. The NH_4_^+^ concentration was determined using a multi-mode microplate reader (M5, Molecular Devices, San Jose, CA, United States). The excitation wavelength was 600 nm, and the signal intensity was measured at an emission maximum of 420 nm.

### Na^+^, K^+^-ATPase Activity

Na^+^/K^+^-ATPase activity was defined as the difference between the concentration of inorganic phosphate liberated in the presence and absence of a protein inhibitor. Ouabain was used as the NKA inhibitor. The inorganic phosphate concentration was determined with microplates according to the method of Peterson (1978). Gills were homogenized (TissueLyser, Qiagen, Germany) for 1 min. Homogenate was mixed with 40 μl of the reaction solution. In the ouabain-treated samples, reagent medium (142.85 mM imidazole, 178.5 mM NaCl, 10.71 mM MgCl_2_, 107.14 mM KCl) was mixed with 10 mM ouabain. In ouabain-free samples, reagent medium was mixed with deionized water. Next, 30 mM Na_2_ATP was pipetted into each well and the reactions were incubated at 37°C. After the addition of Bonting’s color reagent (176 mM FeSO_4_, 560 mM H_2_SO_4_, and 8.1 mM ammonium molybdate tetrahydrate), the mixture was incubated at 20°C. The inorganic phosphate concentration was determined by absorbance spectroscopy (Enzyme Linked ImmunoSorbent Assay, Thermo, United States) at a wavelength of 690 nm.

### Statistical Analysis

R (version 2.15.2) was used for statistical analyses. All values are presented as mean ± SEM. Data were analyzed using a two-way analysis of variance (ANOVA) and Tukey’s HSD test at a 5% level of significance.

## Results

### Air-Breathing Frequency

**FIGURE 1:**
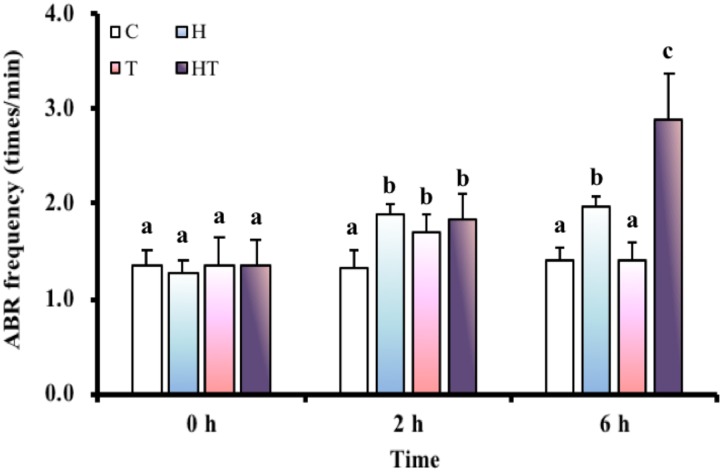
ABR frequency (*n* = 8–12) of *M. opercularis* in control (C), hypoxic (H), thermal (T), and hypoxic-and-thermal (HT) treatments at 0, 2, and 6 h. Values that were significantly different (*p* < 0.05) among treatments and times are indicated by lowercase letters. Data are presented as mean ± SEM.

We first tested whether increased air breathing was a strategy used by *M. opercularis* to respond to both hypoxic and thermal stress. The air-breathing frequency (Figure 1) was significantly increased in H and HT treated fish at 2 h and 6 h, but was only increased by T treatment at 2 h (Table 3, two-way ANOVA; Tukey’s HSD test, H_2__h_: *p* = 0.001, H_6__h_: *p* < 0.001, T_2__h_: *p* = 0.148, HT_2__h_: *p* = 0.01, HT_6__h_: *p* < 0.001). Furthermore, at 6 h, the air-breathing frequency for HT treated fish was higher than that of the fish under H treatment alone (Table 3, two-way ANOVA; Tukey’s HSD test, *p* < 0.001).

**Table 3:** The effects of control (C), hypoxic (H), thermal (T), hypoxic-and-thermal (HT) treatments on 0, 2, and 6 h in ABR group based on two-way ANOVA.

	df	MS	*F*	*P*
**ABR frequency**				
Treatment	3	2.92	49.14	<0.001
Time	2	4.26	71.55	<0.001
Treatment × Time	6	1.90	31.94	<0.001
Error	101	0.06		
**Glucose conc.**				
Treatment	3	9.71	20.79	<0.001
Time	2	21.12	45.25	<0.001
Treatment × Time	6	4.93	10.57	<0.001
Error	108	0.47		
**Lactate conc.**				
Treatment	3	<0.001	0.18	0.91
Time	2	<0.001	1.04	0.36
Treatment × Time	6	<0.001	0.49	0.82
Error	105	<0.001		
**NH_4_^+^ conc.**				
Treatment	3	248.01	37.13	<0.001
Time	2	1671.29	250.20	<0.001
Treatment × Time	6	86.26	12.91	<0.001
Error	78	6.68		
**NKA activity**				
Treatment	3	0.94	0.59	0.62
Time	2	0.73	0.46	0.63
Treatment × Time	6	0.82	0.52	0.79
Error	103	1.59		
**GP protein relative abundance**				
Treatment	3	0.20	1.50	0.22
Time	2	0.23	1.70	0.19
Treatment × Time	6	0.13	0.95	0.46
Error	78	0.14		

### Blood Glucose Concentration

**FIGURE 2:**
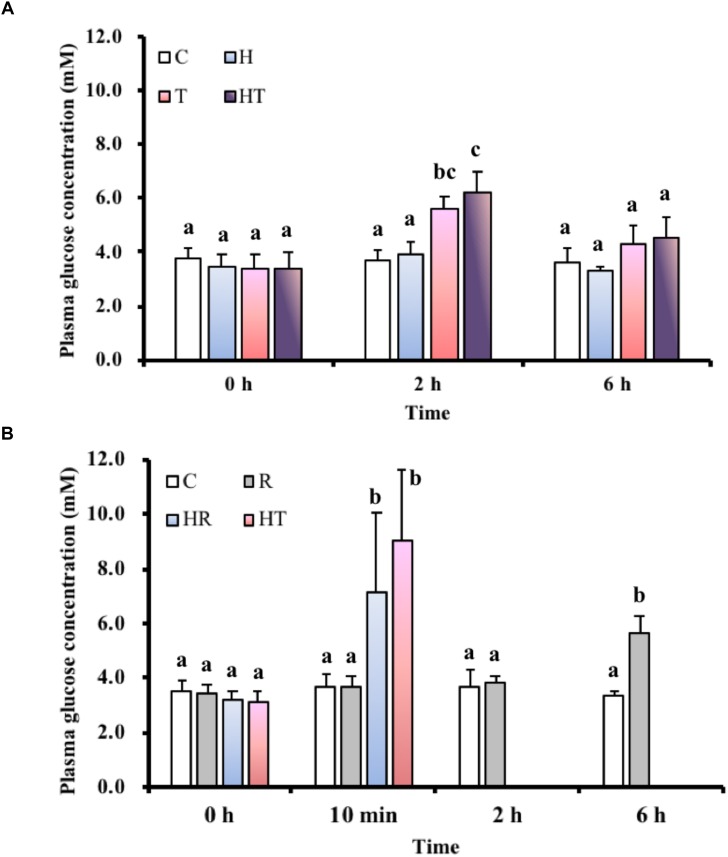
Blood glucose levels in *M. opercularis*: **(A)** in control (C), hypoxic (H), thermal (T), and hypoxic-and-thermal (HT) ABR treatment groups at 0, 2, and 6 h (*n* = 10); **(B)** changes in control (C), restricted (R), hypoxic (HR), and thermal (TR) ABR-restricted treatment groups at 0 h, 10 min, 2 h and 6 h (*n* = 7–10). Values that were significantly different (*p* < 0.05) among treatments and times are indicated by lowercase letters. Data are presented as mean ± SEM.

In the air-breathing group, blood glucose concentrations (Figure 2A) were significantly higher among fish in the T and the HT treatments compared to C treatment at 2 h (Table 3, two-way ANOVA; Tukey’s HSD test, T_2__h_: *p* < 0.001, HT_2__h_: *p* < 0.001). At 6 h, blood glucose concentrations were restored to the same level as at 0 h. In the ABR-restricted group, glucose concentrations (Figure 2B) were significantly higher among fish in the HR and TR treatment than fish in the C treatment group at 10 min (Table 4, two-way ANOVA; Tukey’s HSD test, HR_10__min_: *p* < 0.001, TR_10__min_: *p* < 0.001).

**Table 4:** The effects of control (C), restricted (R), hypoxic (HR), thermal (TR) treatments on 0 h, 10 min, 2 h and 6 h in ABR-restricted group based on two-way ANOVA.

	df	MS	*F*	*p*
**Glucose conc.**				
Treatment	3	15.97	9.76	<0.001
Time	3	30.89	18.88	<0.001
Treatment × Time	5	13.92	8.51	<0.001
Error	59	1.64		
**Lactate conc.**				
Treatment	3	0.03	10.71	<0.001
Time	3	0.03	7.83	<0.001
Treatment × Time	5	0.01	3.89	<0.001
Error	100	0.003		
**NH_4_^+^ conc.**				
Treatment	3	55.08	16.52	<0.001
Time	3	204.20	61.24	<0.001
Treatment × Time	5	3.66	1.10	0.37
Error	65	3.33		

### Lactate Concentration in the Gills

**FIGURE 3:**
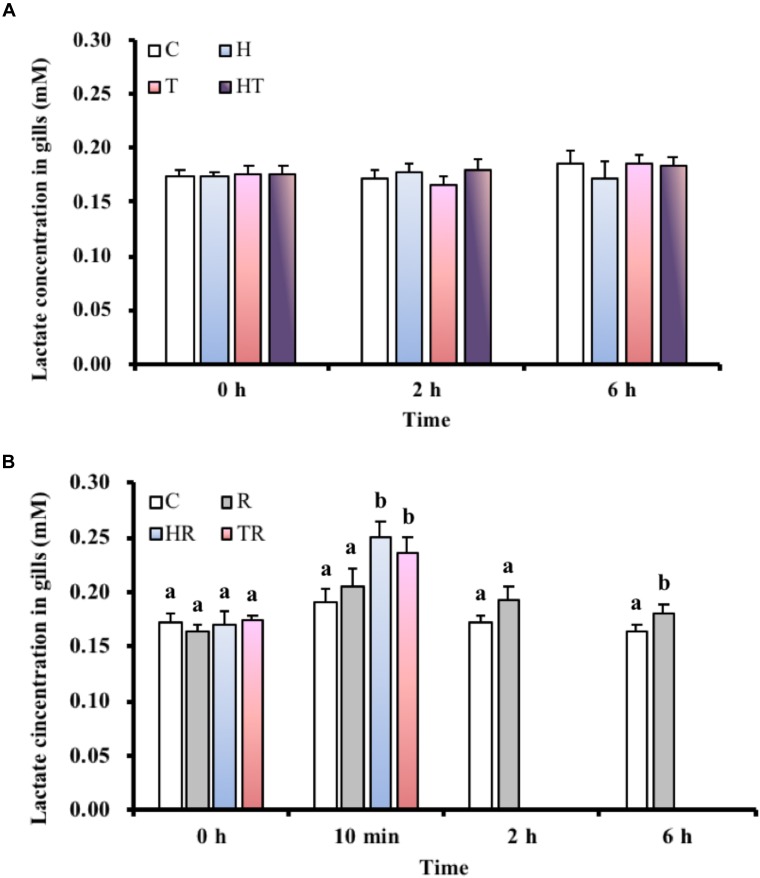
Lactate concentration in gills of *M. opercularis*: **(A)** in control (C), hypoxic (H), thermal (T), and hypoxic-and-thermal (HT) ABR treatment groups at 0, 2, and 6 h (*n* = 9–11); **(B)** in control (C), restricted (R), hypoxic (HR), and thermal (TR) ABR-restricted treatment groups at 0 h, 10 min, 2 h and 6 h (*n* = 7–10). Values that were significantly different (*p* < 0.05) among treatments and times are indicated by lowercase letters. Data are presented as mean ± SEM.

In the air-breathing group, lactate concentrations (Figure 3A) were not significantly different (Table 3, two-way ANOVA) among the different treatments and sampling times. However, the lactate concentrations of fish under the HR and TR treatment (Figure 3B) were significantly higher than those of fish under the C treatment at 10 min in the ABR-restricted group (Table 4, two-way ANOVA; Tukey’s HSD test, HR_10__min_: *p* < 0.001, TR_10__min_: *p* < 0.001).

### Relative Protein Abundance of GP in Gills

**FIGURE 4:**
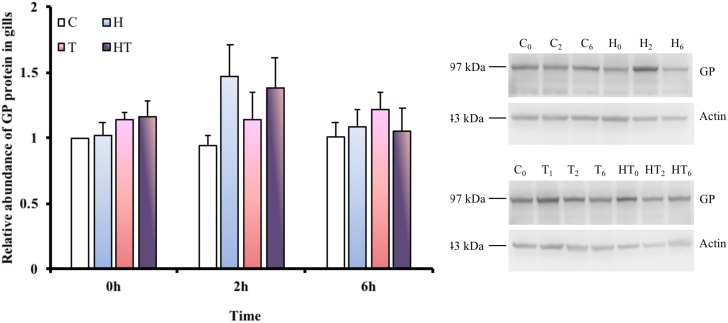
Relative abundance of GP protein was found at approximately 97 kDa in *M. opercularis* in control (C), hypoxic (H), thermal (T), and hypoxic-and-thermal (HT) treatment groups at 0, 2, and 6 h (*n* = 7–9). All GP protein expression of fish were showed by their relative value to C treatment at 0 h. There were no statistically significant differences among treatments and times. Data are presented as mean ± SEM.

In order to test whether glucose mobilization was upregulated by thermal or hypoxic stress, we measured GP protein levels in gill homogenate. Because *M. opercularis* die quickly when air breathing is restricted during times of stress, we only investigated homeostatic changes in glycogen metabolism under conditions that allowed air breathing. A single protein band was detected from the *M. opercularis* gill homogenate by anti-human GPBB, which corresponded to the predicted 97 kDa molecular weight of the protein (Figure 4). No significant differences were observed in GP protein abundance among the different treatments and sampling times (Table 3, two-way ANOVA).

### Concentration of Excreted NH_4_^+^

**FIGURE 5:**
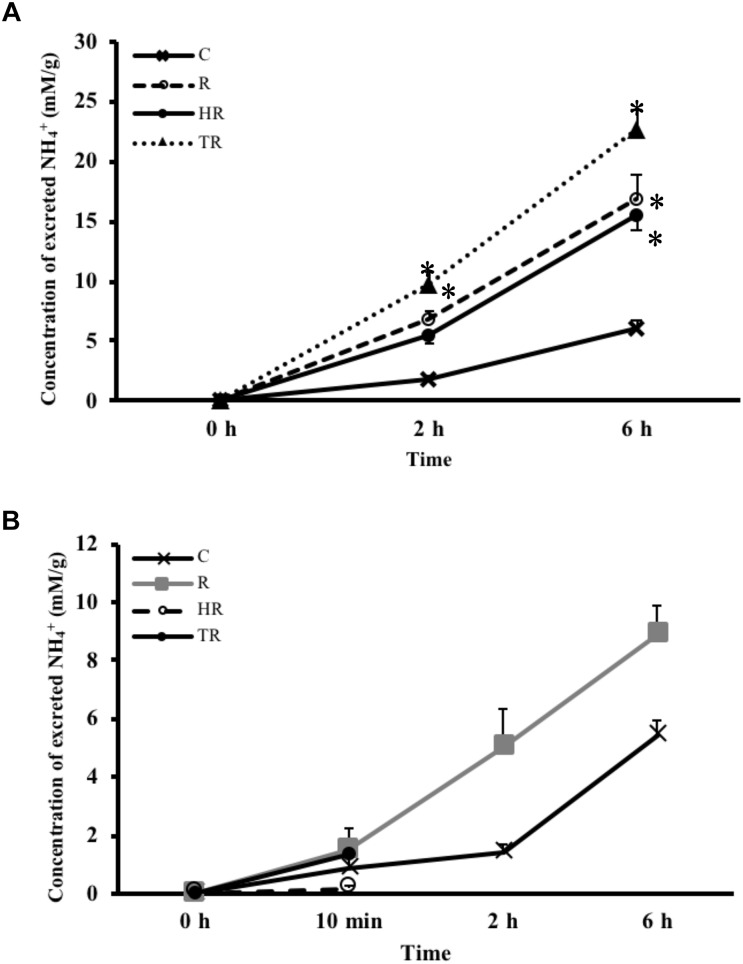
Concentration of excreted NH_4_^+^ from *M. opercularis*: **(A)** in control (C), hypoxic (H), thermal (T), and hypoxic-and-thermal (HT) ABR treatment groups at 0, 2, and 6 h (*n* = 7–9); **(B)** NH_4_^+^ concentrations in control (C), restricted (R), hypoxic (HR), thermal (TR) ABR-restricted treatment groups at 0 h, 10 min, 2 h and 6 h (*n* = 7–9). Values that were significantly different (*p* < 0.05) from control are indicated by an asterisk. There were no statistically significant differences among treatments and times. Data are presented as mean ± SEM.

NH_4_^+^ concentration is not only an indicator of protein metabolism efficiency, but it is also a biomarker of energy expenditure. NH_4_^+^ concentrations in the water samples increased with time in every treatment group (Figure 5A). Compared to the NH_4_^+^ concentration of the C treatment at the same sampling time, the NH_4_^+^ concentration increased under H and HT treatments at 2 h (Table 3, two-way ANOVA, Table 2; H: *p* = 0.010, HT: *p* < 0.001) and increased under the H, T and HT treatments at 6 h (two-way ANOVA; H: *p* < 0.001, T: *p* < 0.001, HT: *p* < 0.001) in the air-breathing group. The NH_4_^+^ concentration under the HT treatment was higher than that observed under the H treatment and T treatment at 6 h (H_6__h_: *p* = 0.004, T_6__h_: *p* < 0.001). In the ABR-restricted group, there was no significant difference in the NH_4_^+^ concentrations (Figure 5B) among the different treatments and sampling times (Table 4, two-way ANOVA). Based on these data, we conclude that NH_4_^+^ concentration increased in concert with air-breathing frequency.

### NKA Activity in the Gills

**FIGURE 6:**
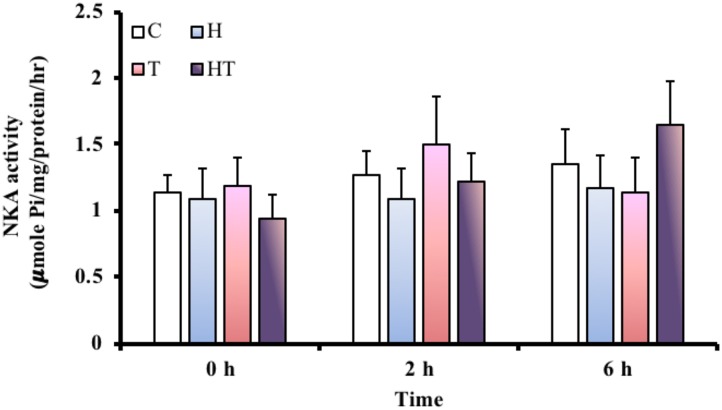
NKA activity in gills of *M. opercularis* in control (C), hypoxic (H), thermal (T), and hypoxic-and-thermal (HT) treatment groups at 0, 2, and 6 h (*n* = 7–9). There were no statistically significant differences among treatments and times. Data are presented as mean ± SEM.

Because ionoregulation is a major energy expenditure, we measured NKA activity to better understand how *M. opercularis* regulates energy consumption under stress. Compared to the NKA activity in the C treatment group at each sampling time, the specific activity of NKA in the gills (Figure 6) did not differ. Similarly, the specific activity of NKA in the gills did not differ significantly among the different sampling times (Table 3, two-way ANOVA). Thus, the fish did not decrease energy expenditure by inhibiting NKA activity in response to hypoxic or thermal stresses.

## Discussion

*M. opercularis* is an air breather, which performs aerial respiration in both normoxic and hypoxic conditions. However, according to our mortality data, air breathing is a necessary behavior that allows *M. opercularis* to compensate the environmental hypoxia and thermal stress. Blood glucose is an important fuel that is often elevated to support organisms during stressful conditions (Wells and Pankhurst, 1999; Nelson and Cox, 2007). However, blood glucose may not be a reliable indicator of stress with regard to air-breathing *M. opercularis*. According to Huang et al. (2015a), NKA and carbonic anhydrase II protein levels were changed in fourth gill when the oxygen level was below 1.85 ± 0.37 mg/l, indicating that *M. opercularis* may be stressed below this oxygen level. In our study, the hypoxic conditions were well below this threshold. Interestingly, the blood glucose concentration of *M. opercularis* did not change under hypoxia in the ABR group. Since ABR provides direct access to oxygen, an increase in the ABR frequency is expected to compensate for limited oxygen supply in aquatic hypoxic conditions (Graham, 1997; Takasusuki et al., 1998; Timmerman and Chapman, 2004; Affonso and Rantin, 2005; MacCormack et al., 2006; Richards et al., 2009). The same behavioral compensation to low oxygen levels has been observed by our laboratory in two other air-breathing species, *Helostoma temminckii* and *Trichogaster microlepis* (Huang et al., 2015b; Lin and Lin, unpublished data). Thus, ABR frequency may be a better indicator of hypoxic defense than blood glucose concentration in anabantoid fish. Based on the above reasons, increased ABR frequency in H, T, and HT treatments was an indication of the presence of the hypoxic stress. Increased ABR behavior may temporarily satisfy oxygen demand while further adjustments are made at the cellular level.

In hypoxia-tolerant fishes, including *Astronotus ocellatus*, *Cyprinus carpio*, and *Liposarcus pardalis*, tissue glycogen is broken down to glucose to provide fuel for lactate-producing anaerobic metabolism (Zhou et al., 2000; MacCormack et al., 2006; Wood et al., 2007; Genz et al., 2013). These observations suggest that an oxyconforming strategy is used in hypoxia-tolerant water-breathing fishes, wherein physical activities, aerobic metabolism rates and energy demands all decrease as the partial pressure of oxygen decreases. Conversely, a fish with an oxyregulating strategy would maintain physical activity, aerobic metabolism rate and energy requirements during hypoxia, until a critical partial pressure of oxygen is reached. We made contrasting observations in hypoxia-tolerant air-breathing *M. opercularis*, which suggest the fish does not strictly adhere to an oxyconforming or oxyregulating strategy. Increasing the frequency of ABR was critical to maintain oxygen uptake and energy homeostasis in *M. opercularis*. According to the unchanged GP protein expression and lactate concentration in gill, *M. opercularis* behaves as an oxyregulator to maintain its aerobic metabolism rate under hypoxic conditions by utilizing ABR. Conversely, on the basis of our combined lactate and glucose data, an anaerobic operation arose in the gill at 6 h after air-breath restriction. And it is possible the fish could require increased mobilization of glucose to fuel anaerobic metabolism. It represents *M. opercularis* acts as an oxyconformer and as a hypoxia-intolerant fish when it cannot perform ABR. In this study, we found that although *M. opercularis* can survive when air breathing is restricted as a non-obligatory continuous air breather, as described by Graham (1997), it would be an oxyconformer under non-stressed conditions. Metabolites reorganization and alterations in efficiency of different metabolic pathway happen in changing environment (Evans and Claiborne, 2006; Costas et al., 2011). The increased NH4^+^ shows an increased amino acid metabolic rate under hypoxic and thermal stresses. However, NH_4_^+^ is toxic to animal tissues. Fish excretes NH_4_^+^ product by ammonia (NH_3_) form to the water, and NH_3_ usually is accompanied with an excretion of protons (or a proton in ambient) and converts to NH_4_^+^ form (Hwang et al., 2011). Since excretion is such a metabolically expensive process, some fishes will downregulate amino acid metabolism to minimize NH_4_^+^ production during adverse conditions. Alternatively, the fish may accumulate NH_4_^+^ in the body (Ip et al., 2004a,b; Val et al., 2005; Randall and Ip, 2006; Wood et al., 2007). For example, *Protopterus annectens*, an obligatory air-breather, responds to prolonged air exposure by increasing conversion of ammonia to urea and decreasing ammonia production (Loong et al., 2008). In the hypoxia-tolerant water-breathing Amazonian cichlid, *Astronotus ocellatus*, plasma ammonia was reported to be increased as the ammonia excretion rate decreased in a 3 h hypoxic exposure. It was suggested that *A. ocellatus* reduced its ammonia excretion rate to conserve energy under hypoxia, leading to the accumulation of ammonia (Wood et al., 2007). On the contrary, we found that NH_4_^+^ excretion from *M. opercularis* increased under hypoxic and thermal stress in the ABR group but not under ABR-restricted treatment. The difference might result from the increased ABR frequency, which is known to enhance the rate of adenylate deamination in fish muscle and thereby produce NH_4_^+^ (Val et al., 2005; Moyes and Schulte, 2008). Indeed, we didn’t measure the NH_4_^+^ accumulation in fish body for understanding the NH_4_^+^ production rate of fish. However, the increased NH_4_^+^ excretion in water represents a raised amino acid metabolic rate, and, the NH_4_^+^ accumulation in *M. opercularis* may exceed its tolerance under hypoxia and thermal stresses. To determine the NH_4_^+^ production of muscle would be a further work for a more direct connection of ABR frequency and amino acid metabolism.

To conserve energy expenditure, NKA activity was diminished in *A. ocellatus* gill by 60% when exposed to hypoxia for 3 h (Wood et al., 2007). In a similar result, the gill NKA activity of *Platichthys flesus* was decreased by approximately 25% after 48 h of hypoxia (Lundgreen et al., 2008). In contrast, decreased NKA activity was not observed in our results with *M. opercularis*, as it remained unchanged at 6 h under all treatments in the ABR group. Neither thermal nor hypoxic stresses attenuate NKA activity in the hypoxia-tolerant *Carassius auratus* (Mitrovic and Perry, 2008; Mitrovic et al., 2009). The authors proposed that *C. auratus* may enlarge the gill surface area to absorb more oxygen and meet oxygen demand during hypoxic and thermal stresses. In fact, NKA activity was increased to maintain ion homeostasis when the enlarged gill surface area allowed more ions to enter the gill tissue (Mitrovic and Perry, 2008; Mitrovic et al., 2009). Anabantoid fishes have also been reported to modify gill morphology or increase MR-cell numbers within the gills under various stresses (Huang et al., 2008, 2011, 2015a,c). As a result, *M. opercularis* may need to maintain NKA activity in order to perform ionoregulation during gill remodeling in addition to supporting general physiological processes. The increased ABR frequency is expected to provide sufficient oxygen to maintain energy levels for NKA operation.

## Conclusion

In conclusion, our results identify that ABR behavior play a critical role in both hypoxic and thermal resistance. Thermal-resistance strategy is similar to the hypoxia-resistance strategy on *M. opercularis.* However, hypoxic stress leads to a decrease in metabolism rate, while, thermal situation causes a rise in metabolic rates (Diaz, 2001; Barnes et al., 2011). The increased glucose mobilized for energy-consuming mechanism only appeared under thermal stress implies that different response mechanisms are activated for the two stressors. The hypoxia-resistance strategy of *M. opercularis* is different from those reported for water-breathing fish. Upon acute situation, the ability to upregulate ABR behavior provides air-breathing fish a unique method for thermal and hypoxia resistance that is unavailable to water-breathing fish. However, the understanding of fish responses in long-term acclimation (on the order of days or weeks) need to be improved in the further study. In any case, ABR behavior is energy-costing. If air-breathing *M. opercularis* uses other mechanism to replace with the behavior for saving energy fuel under long-term environmental stress, for instance, the trade-off of efficiency of different metabolism pathways and the functional/morphological change of ionocytes should be examined with that in the water-breathing fish.

## Ethics Statement

Experimental protocols were approved by the Tunghai University (Taichung City, Taiwan) (approval no. 99-18).

## Author Contributions

M-CW and H-CL designed the experiments and wrote the first draft of the manuscript. M-CW conducted the analysis.

## Conflict of Interest Statement

The authors declare that the research was conducted in the absence of any commercial or financial relationships that could be construed as a potential conflict of interest.
